# Supplementary material for: Spatiotemporally-resolved mapping of RNA binding proteins via functional proximity labeling reveals a mitochondrial mRNA anchor promoting stress recovery
Source: Nat Commun. 2021 Aug 17;12:4980. doi: 10.1038/s41467-021-25259-2 (PMC8370977; doi:10.1038/s41467-021-25259-2)
Supplement: Supplementary file 1 — Supporting information [file 41467_2021_25259_MOESM1_ESM.pdf]

Supplementary Information

Spatiotemporally-resolved mapping of RNA binding proteins via functional proximity labeling reveals a mitochondrial mRNA anchor promoting stress recovery

Wei Qin[1,2], Samuel A Myers[3,4], Dominique K. Carey[3], Steven A Carr[3], Alice Y Ting[1,2*],

[1]Departments of Biology, Genetics, and Chemistry, Stanford University, Stanford, CA, USA; [2]Chan Zuckerberg Biohub, San Francisco, CA, USA; [3]The Broad Institute of MIT and Harvard, Cambridge, MA, USA. [4]La Jolla Institute for Immunology, La Jolla, CA, USA.

[*]Correspondence to: ayting@stanford.edu

This file includes Supplementary Figures 1-12 and Supplementary Tables 1

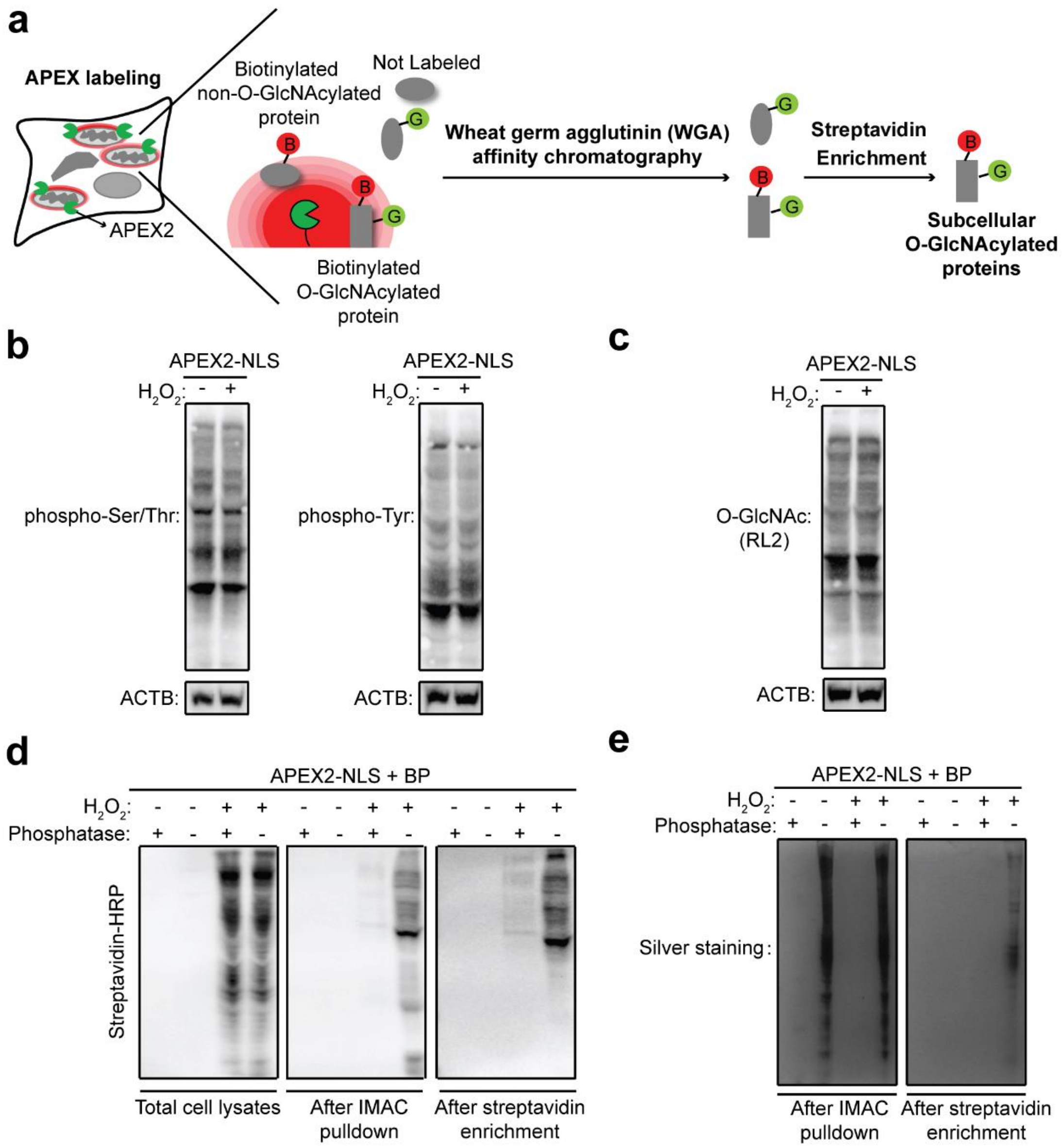

Supplementary Figure 1. Development and validation of functional proximity labeling to study subcellular phosphorylation and O-GlcNAcylation. a, Procedure combining APEX-catalyzed PL with WGA affinity chromatography to enrich subcellular O-GlcNAcylated proteins. Red B, biotin. Green G, O-GlcNAcylation. **b**, The impact of 1 min H_2O_2 treatment (used for APEX labeling) on global phosphor-serine/threonine (left) and phosphotyrosine (right) levels. **c**, The impact of 1 min H_2O_2 treatment on global O-GlcNAcylation levels. The O-GlcNAc antibody used is RL2. **d**, Streptavidin blotting reveals enrichment of nuclear phosphoproteins by APEX-IMAC. Biotinylation was performed in HEK293T cells expressing nuclear-localized APEX (APEX-NLS). Streptavidin blotting was performed on total cell lysate (left), samples after IMAC enrichment (middle), and samples after both IMAC and streptavidin enrichment (right). **e**, Silver staining of enriched nuclear phosphoproteins.

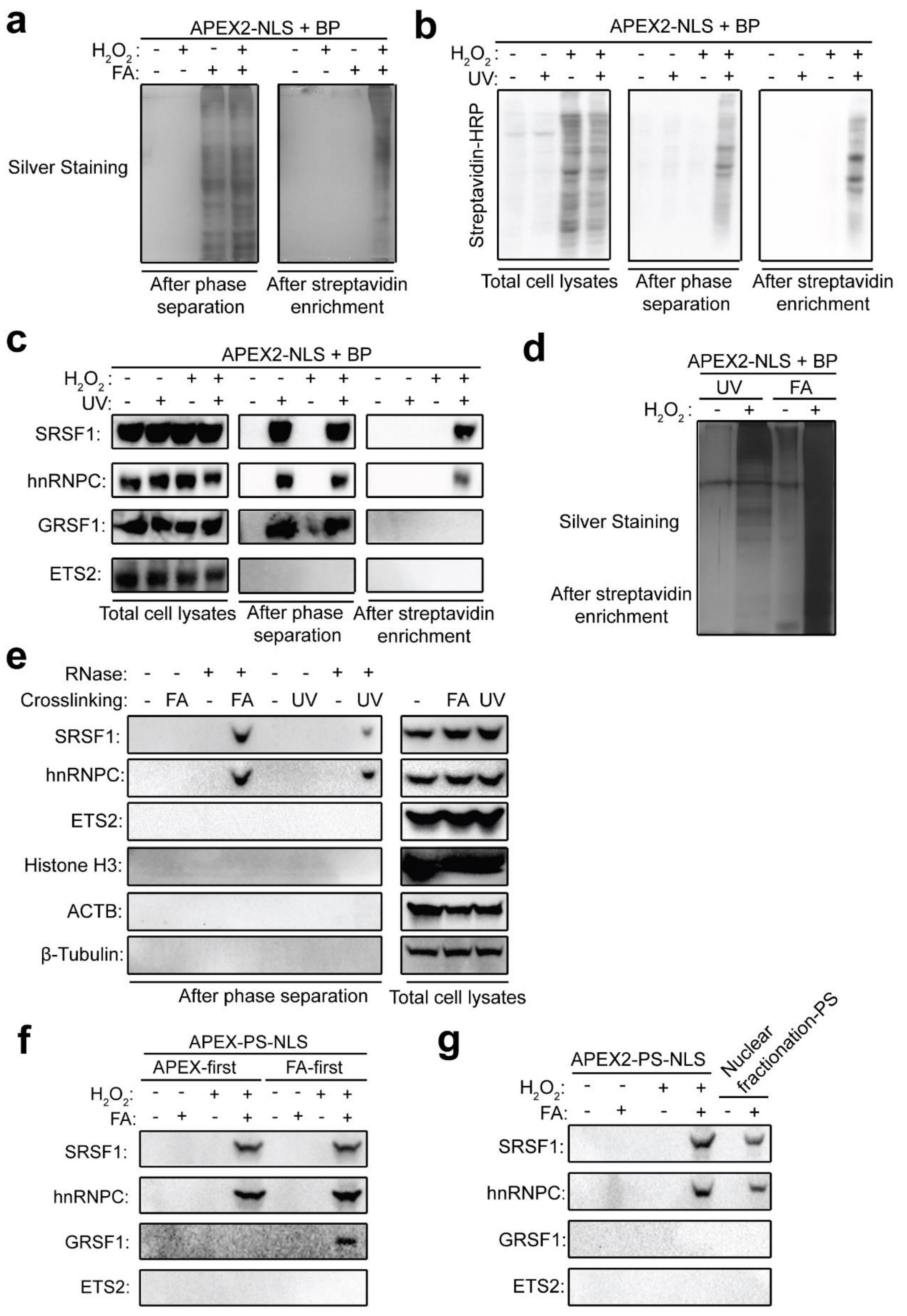

Supplementary Figure 2. Development and characterization of APEX-PS for subcellular RBP enrichment. a, Silver staining shows the enrichment of subcellular RBPs. HEK cells stably expressing APEX-NLS were labeled with biotin-phenol and crosslinked with formaldehyde as shown in Fig. 2a. Lysates were analyzed after phase separation (left) or after both phase separation and streptavidin bead enrichment

(right). **b,** Streptavidin blotting reveals enrichment of nuclear RBPs by APEX-PS with UV crosslinking. Same as Fig. 2c, but with UV crosslinking (400 mJ/cm^2 at 254 nm) instead of formaldehyde crosslinking. **c,** Western blot detection of known nuclear RBPs in samples from (**b**). Same as Fig. 2d, but with samples prepared using UV-crosslinking based APEX-PS. **d,** Comparison of FA- and UV-based APEX-PS. HEK cells expressing APEX-NLS were labeled and crosslinked as shown in Fig. 2a. After phase separation and streptavidin enrichment, eluates were analyzed by silver staining. FA crosslinking recovers much more material than UV crosslinking. **e,** Comparison of FA and UV crosslinking in OOPS (no APEX labeling or streptavidin enrichment). FA or UV-treated HEK cells were subjected to three rounds of phase separation and the interphase was treated with RNase to release crosslinked proteins. After another round of phase separation, proteins in the organic phase were blotted with the antibodies shown. SRSF1 and hnRNPC are known nuclear RBPs. ETS2, Histone H3 are nuclear non-RBPs, and ACTB and β-Tubulin are non-nuclear non-RBPs. **f,** Comparison of APEX-then-FA and FA-then-APEX protocols for enriching nuclear RBPs by APEX-PS. The enriched material was blotted with the antibodies used in Fig. 2d. **g,** Comparison of nuclear APEX-PS and nuclear fractionation followed by OOPS (nuclear fractionation-PS). Both methods start with ~$5x10^7$ cells and the enriched material was blotted with the antibodies used in Fig. 2d.

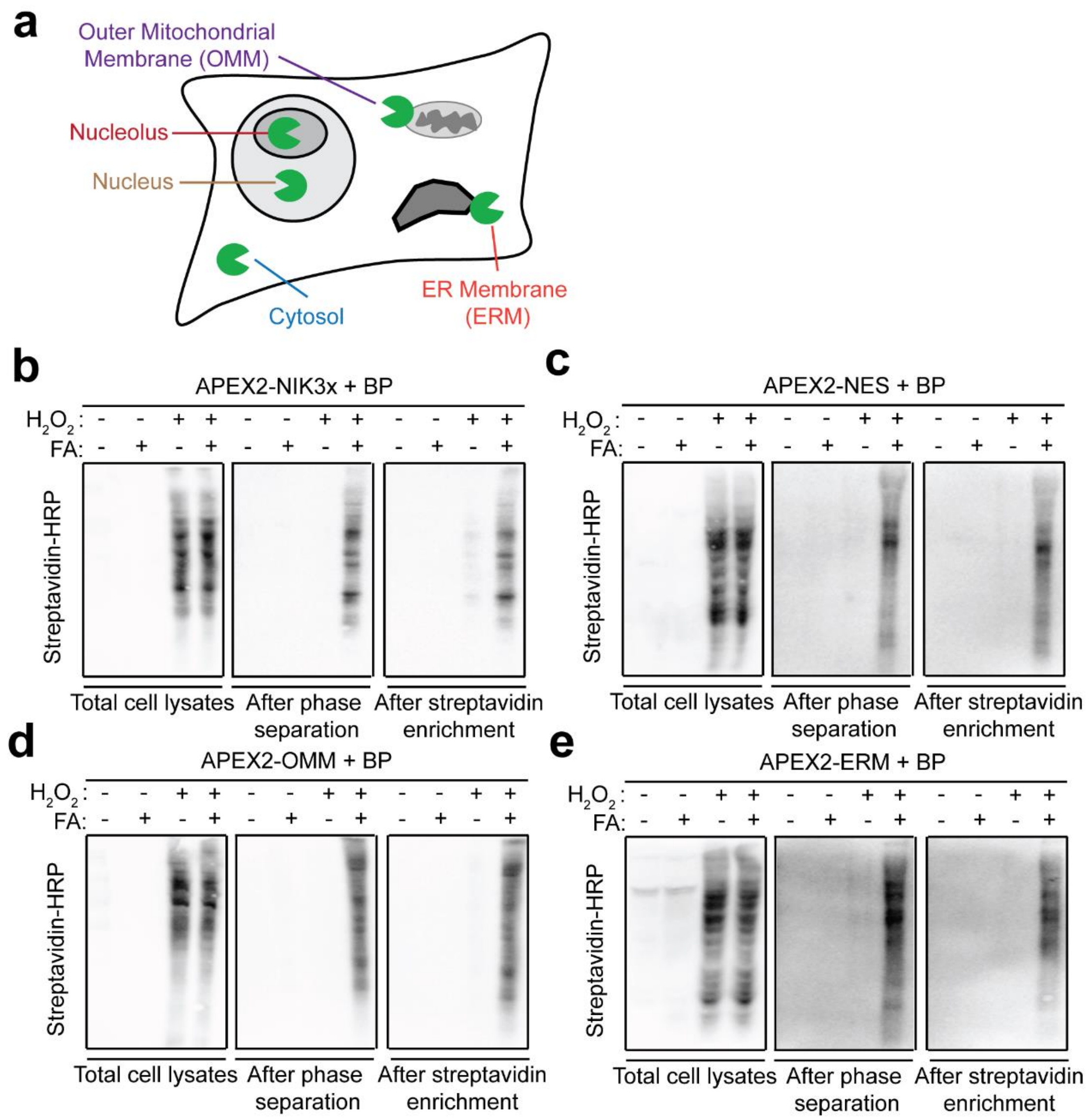

Supplementary Figure 3. Evaluation of APEX-PS in different subcellular compartments. a, Schematic of five subcellular regions tested. (**b-e**) Evaluation of APEX-PS in the nucleolus (**b**), cytosol (**c**), OMM (**d**) and ER membrane (ERM) (**e**) following the procedure shown in Fig. 2a-b.

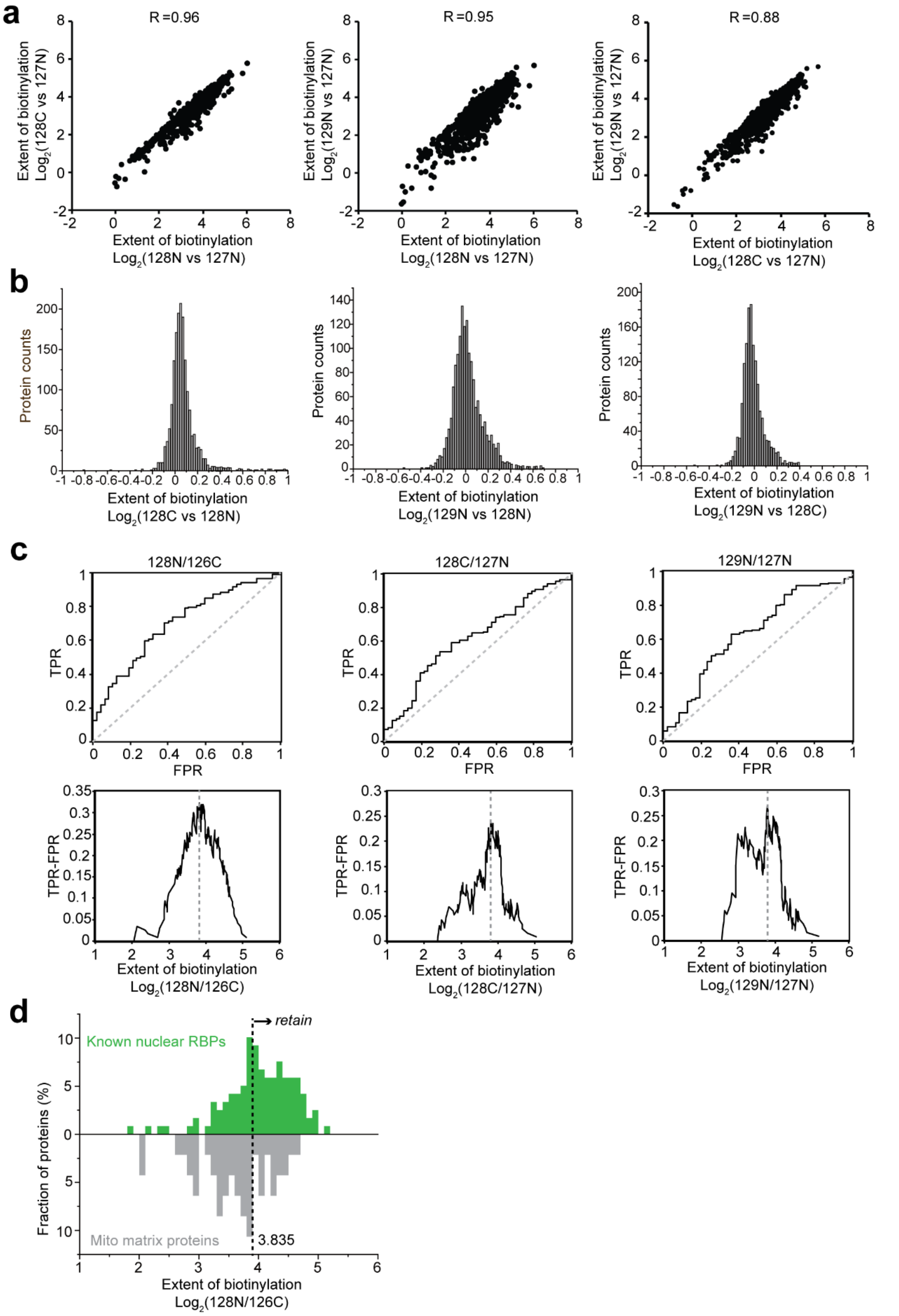

Supplementary Figure 4. Generation of nuclear APEX-PS dataset by pairwise ROC-based filtering. a, Correlation between biological replicates. TMT labels refer to samples in Fig. 3a. **b,** Histograms showing normal distributions of TMT ratios across three biological replicates. **c,** Receiver operating characteristic (ROC) curves of

TMT ratios used for assignment of nuclear RBPs. Proteins were ranked in descending order based on the TMT ratio. True positive denotes known nuclear RBPs (Supplementary Table 1, tab 2). False positives denote mitochondrial matrix proteins (Supplementary Table 1, tab 2). **d,** Sample histogram showing how the cutoff for 128N/126C ratio was applied.

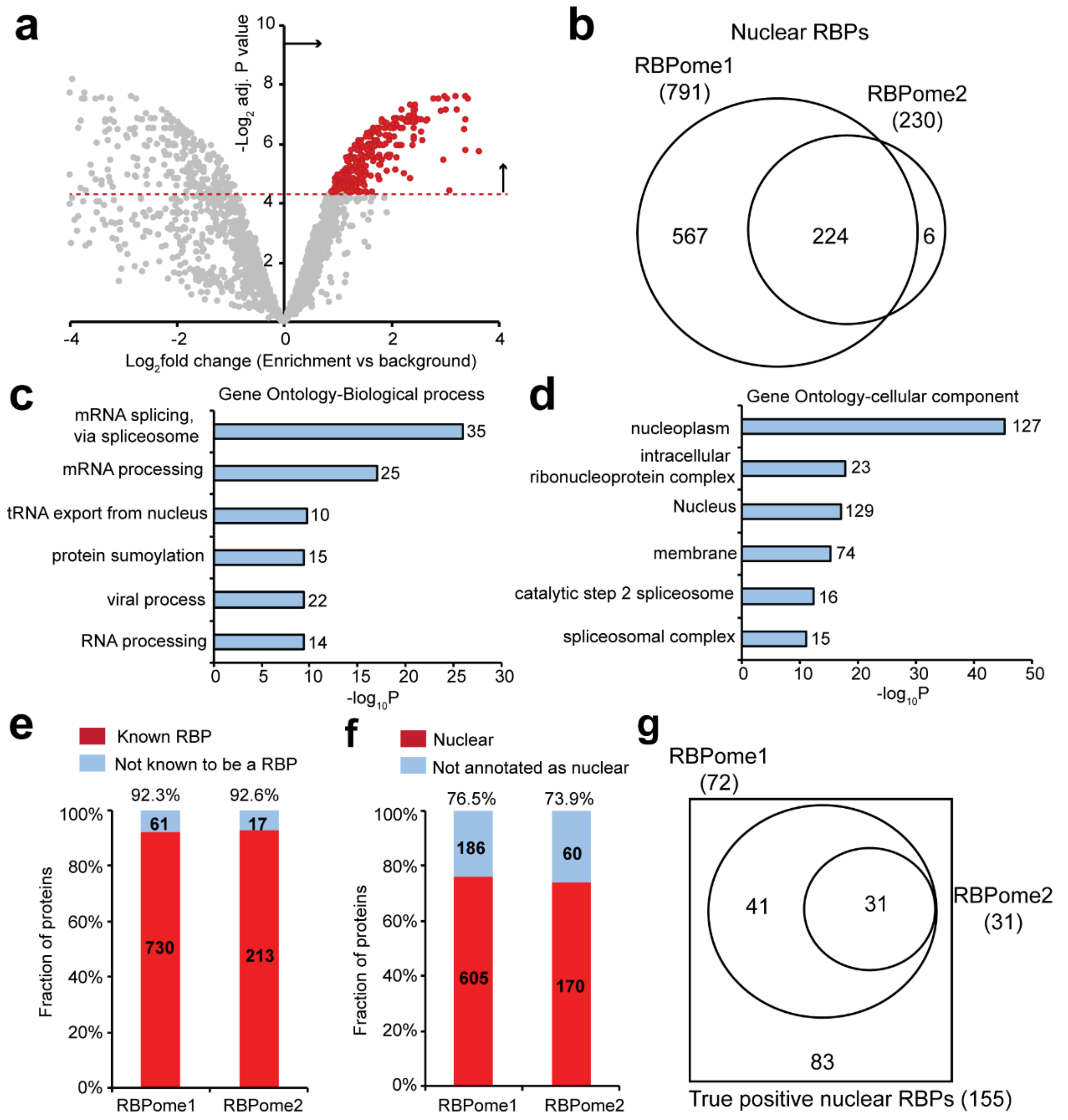

Supplementary Figure 5. Generation of nuclear APEX-PS dataset by statistical analysis. **a,** Volcano plot of proteins quantified in APEX-PS-NLS replicates compared to the background controls. Red data points represent proteins enriched with an adjusted p-value of less than 0.05 and fold change of enrichment versus background greater than 1 (Supplementary Table 2, tab 2). **b,** Venn diagram comparing nuclear RBPomes generated by pairwise ROC analysis (nuclear RBPome1) and statistical analysis (nuclear RBPome2). **c,** GOCC analysis of nuclear RBPome2. **d,** GO biological process analysis of nuclear RBPome2. The number of proteins in each GO item is shown. **e,** RBP specificity of nuclear RBPomes. **f,** Nuclear specificity of nuclear RBPomes. **g,** Using a list of 155 gold standard nuclear RBPs (Supplementary Table 2, tab 4), the coverage of the two nuclear RBPomes were compared.

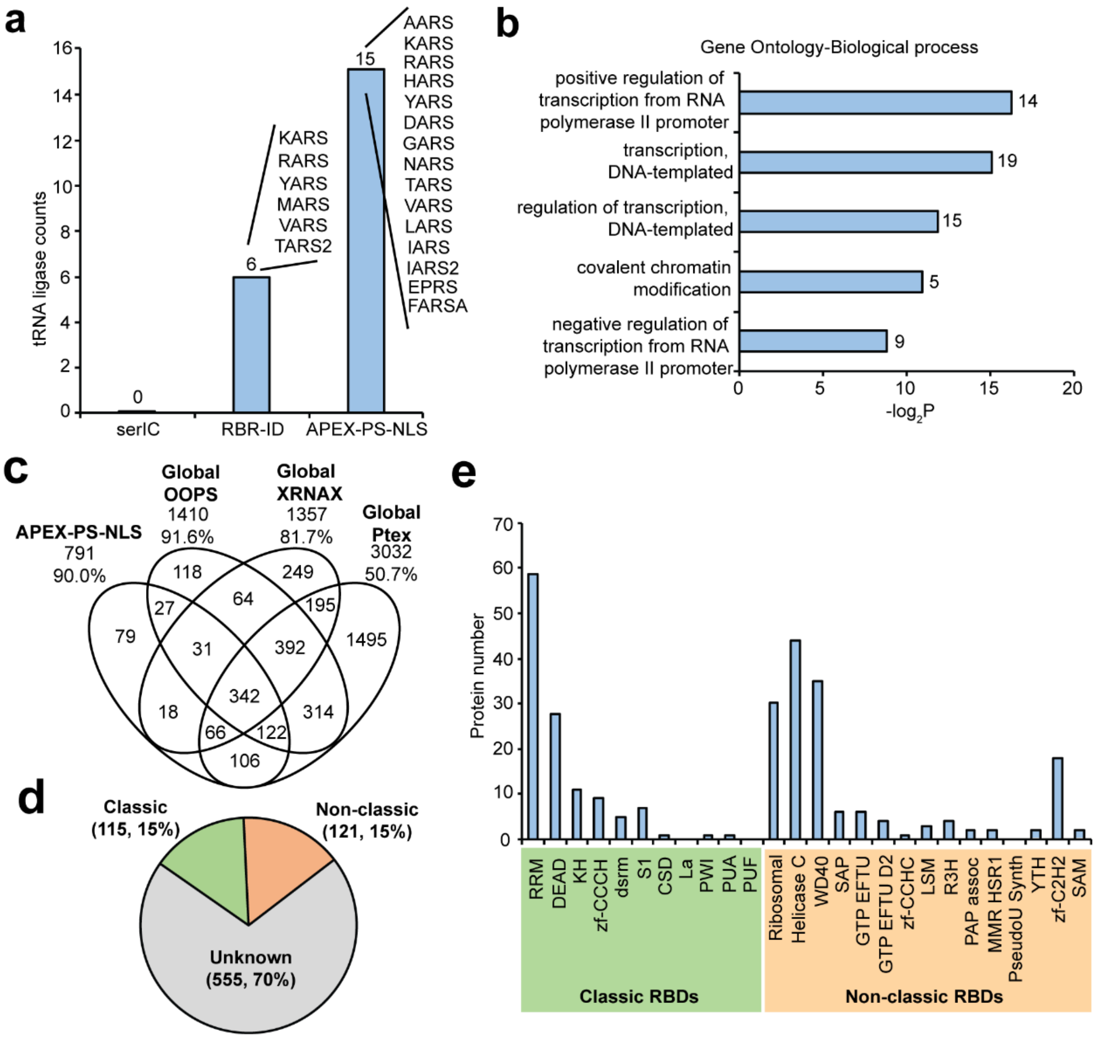

Supplementary Figure 6. Additional analysis of nuclear RBPome1. a, tRNA ligases enriched by APEX-PS versus fractionation-based methods serIC[43] and RBR-ID[44]. **b,** GO biological process analysis of nuclear RBP orphans identified by APEX-PS. **c,** Comparison of nuclear RBPs identified by APEX-PS with other global PS-based RBP profiling strategies, including OOPS[26], XRNAX[27] and Ptex[28]. Under each method is shown the number of proteins in the dataset and the percentage of the dataset that overlaps with one or more other methods. **d,** Percentage of APEX-PS-identified nuclear RBPs with classic versus non-classic RNA binding domains (RBDs). **e,** Number of proteins with each type of classical or non-classical RBD. The RBDs of each RBP are listed in Supplementary Table 2, tab 1.

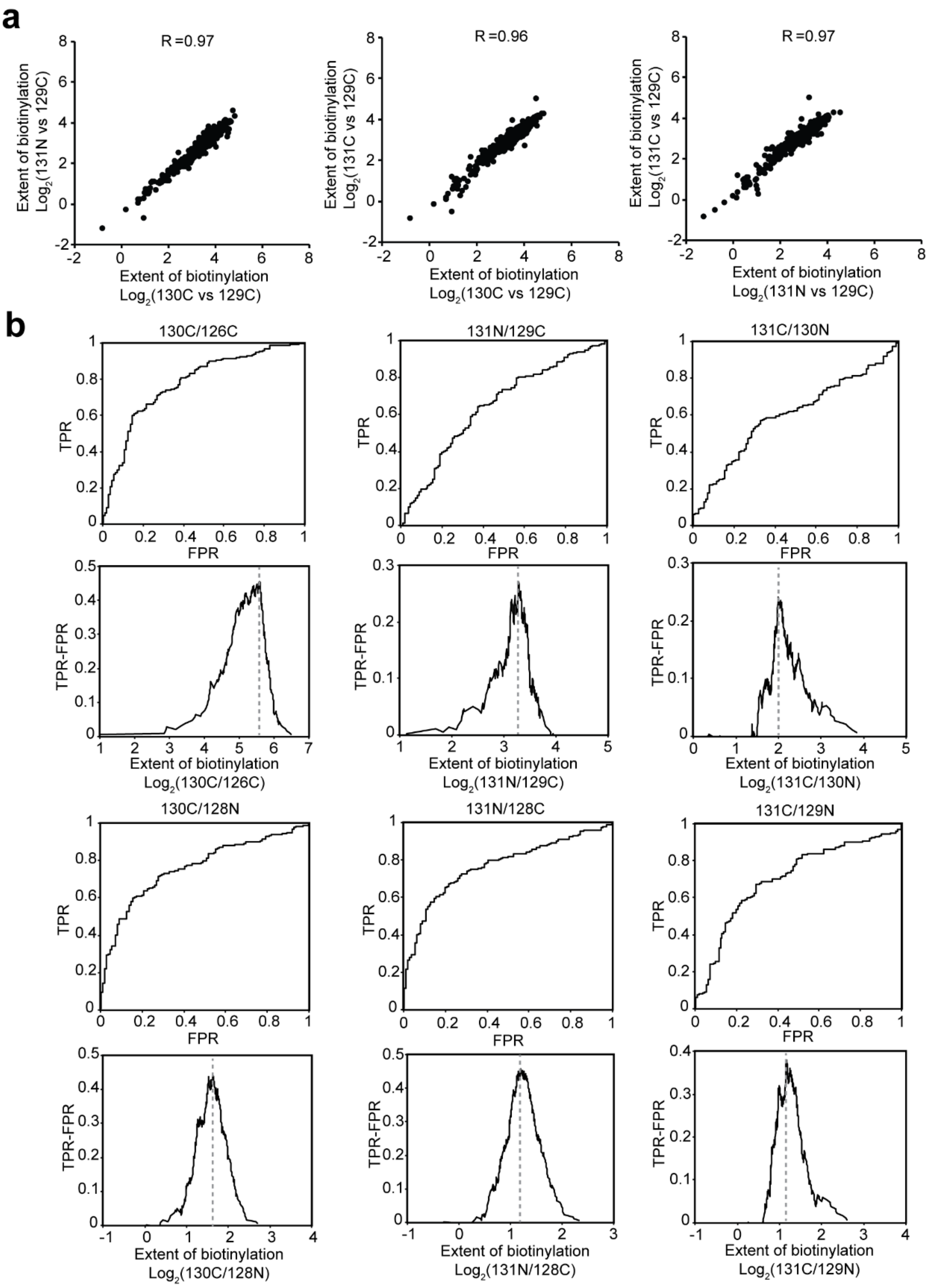

Supplementary Figure 7. Generation of nucleolar APEX-PS dataset by pairwise ROC-based filtering. **a,** Correlation between biological replicates. TMT labels refer to samples in Fig. 3a. **b**, Receiver operating characteristic (ROC) curve of TMT ratios used for assignment of nucleolar RBPs. Proteins were ranked in descending order based on the TMT ratio. True positive denotes known nucleolar RBPs, based on intersecting OOPS RBP datasets[26] with nucleolar GOCC annotation (Supplementary Table 1, tab 3). False positives denote non-nuclear proteins not previously annotated by OOPS datasets (Supplementary Table 1, tab 3).

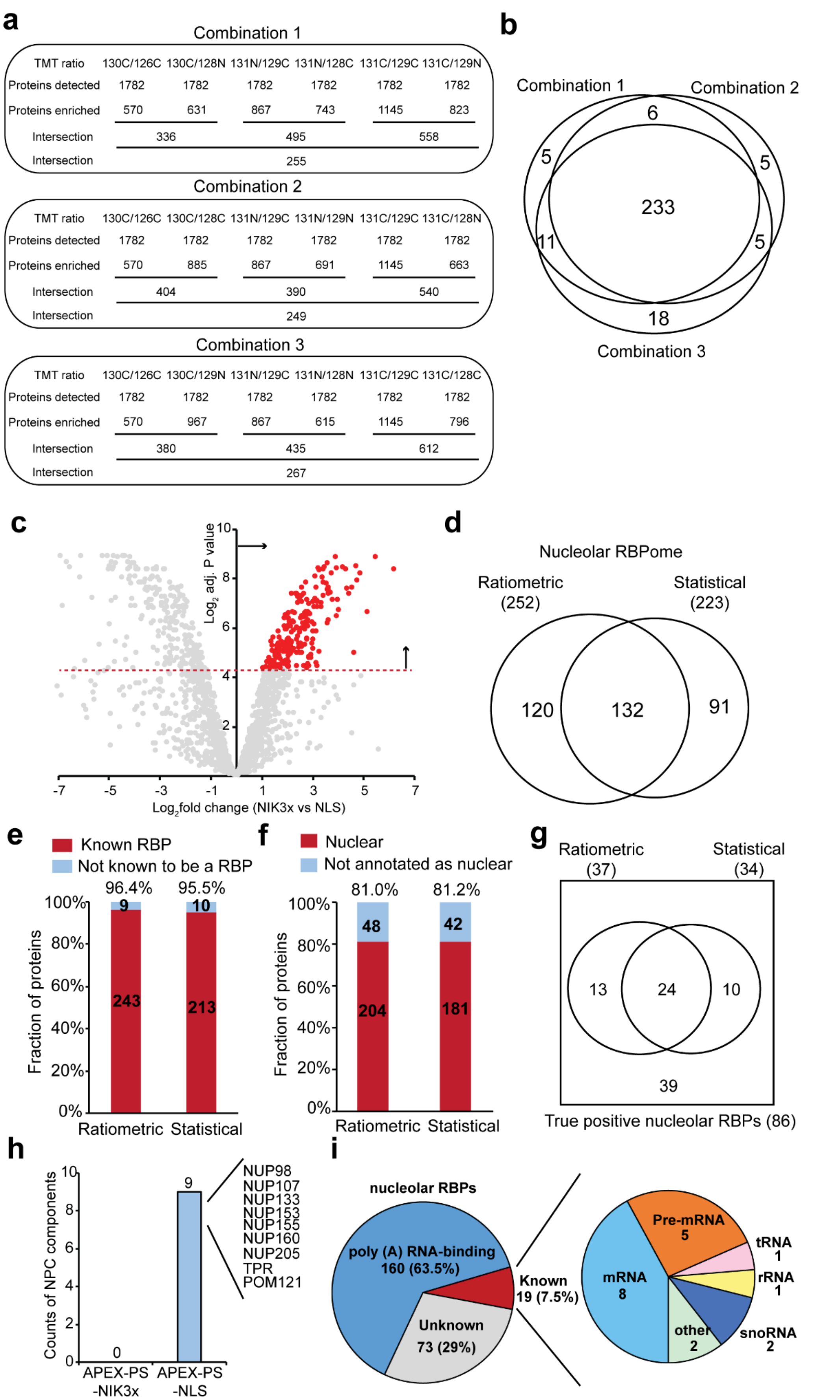

Supplementary Figure 8. Additional analyses of nucleolar APEX-PS dataset. **a,** Nucleolar RBPomes generated by different pairwise combinations of APEX-NIK3x

and APEX-NLS samples. Combination 1 was used to generate our final nucleolar RBPome. **b,** Overlap between datasets generated by Combinations 1-3 in (a). **c,** Volcano plot from statistical analysis and filtering of APEX-PS-NIK3x data. Red data points represent proteins with adjusted p-value < 0.05 and fold change (NIK3x versus NLS) > 1. The protein list is shown in Supplementary Table 3, tab 2. **d,** Venn diagram of nucleolar RBPomes generated from pairwise ROC approach versus statistical analysis. **e,** RBP specificity of nucleolar RBPomes. **f,** Nuclear specificity of nucleolar RBPomes. **g,** Sensitivity analysis using a "gold positive" list of 86 well-established nucleolar RBPs (Supplementary Table 3, tab 3). **h,** The number of nuclear pore complex (NPC) components identified by APEX-PS-NIK3x versus APEX-PS-NLS. **i,** Subclassification of RBPs in nucleolar RBPome1. Many RBPs we enriched bind to poly(A). Of the remainder, 19 have been experimentally shown to bind to the 6 RNA classes shown at right. Further details in Supplementary Table 3, tab1.

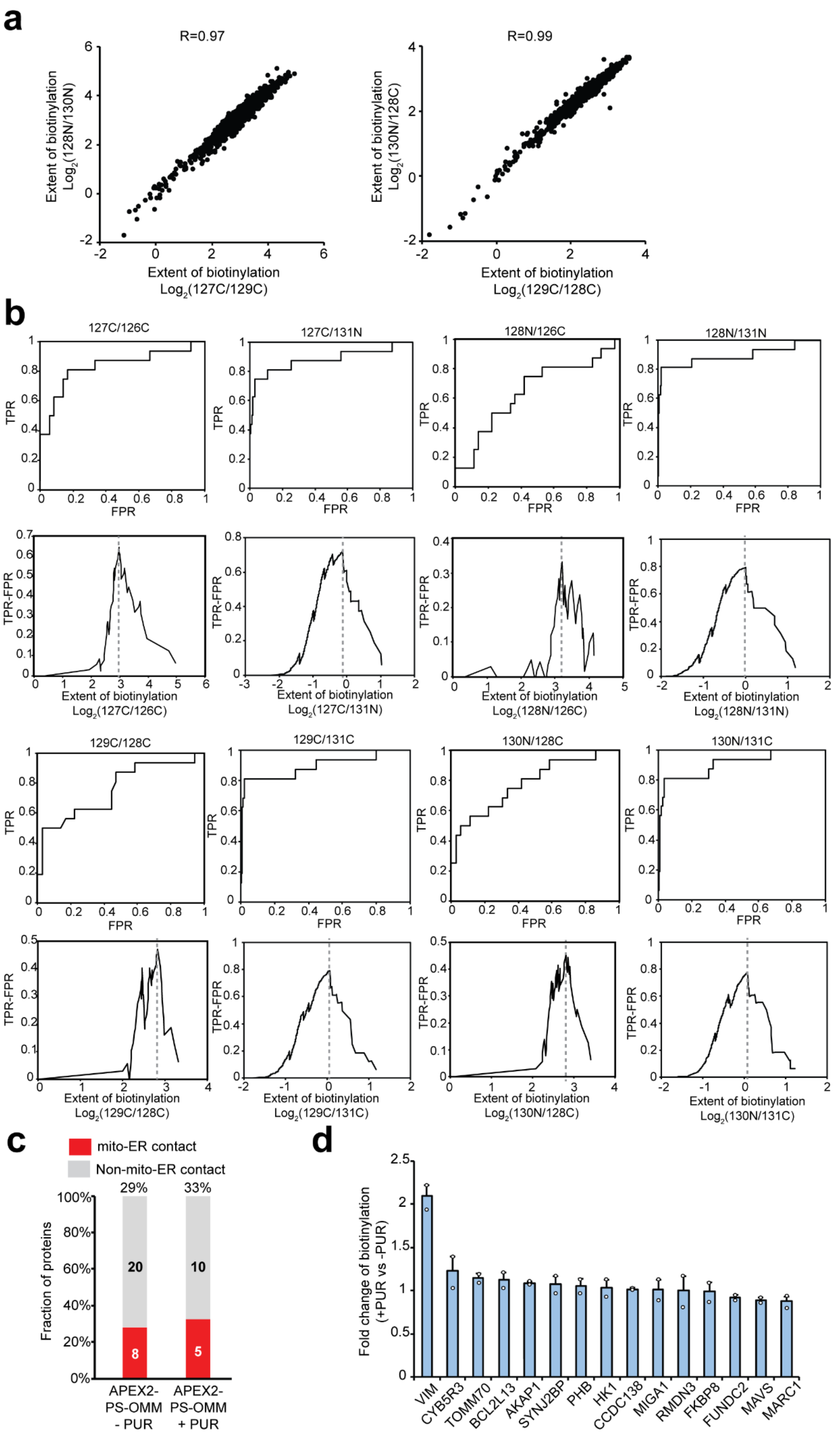

a
R=0.97
Extent of biotinylation Log2(128N/130N)
Extent of biotinylation Log2(127C/129C)
R=0.99
Extent of biotinylation Log2(130N/128C)
Extent of biotinylation Log2(129C/128C)
b
127C/126C
127C/131N
128N/126C
128N/131N
TPR
FPR
TPR-FPR
Extent of biotinylation Log2(127C/126C)
Extent of biotinylation Log2(127C/131N)
Extent of biotinylation Log2(128N/126C)
Extent of biotinylation Log2(128N/131N)
129C/128C
129C/131C
130N/128C
130N/131C
Extent of biotinylation Log2(129C/128C)
Extent of biotinylation Log2(129C/131C)
Extent of biotinylation Log2(130N/128C)
Extent of biotinylation Log2(130N/131C)
c
mito-ER contact
Non-mito-ER contact
Fraction of proteins
29%
33%
20
10
8
5
APEX2-PS-OMM - PUR
APEX2-PS-OMM + PUR
d
Fold change of biotinylation (+PUR vs -PUR)
VIM
CYB5R3
TOMM70
BCL2L13
AKAP1
SYNJ2BP
PHB
HK1
CCDC138
MIGA1
RMDN3
FKBP8
FUNDC2
MAVS
MARC1

Supplementary Figure 9. Generation of OMM APEX-PS datasets by pairwise ROC-based filtering. **a,** Correlation between biological replicates for APEX-PS-OMM profiling. TMT labels refer to samples in Fig. 5a. **b,** Receiver operating characteristic (ROC) curve of TMT ratios used for assignment of OMM-localized RBPs. Proteins were ranked in descending order based on the TMT ratio. True positive denotes known OMM proteins (Supplementary Table 4, tab 2). To compare with background controls, false positive proteins are mitochondrial matrix proteins annotated by GOCC (Supplementary Table 4, tab 2). To compare with APEX-PS-NES reference controls, false positive proteins are non-mitochondrial cytosolic proteins annotated by GOCC (Supplementary Table 4, tab 2). **c,** Fraction of OMM-localized RBPs involved in mitochondria-ER contact sites, according to split-TurboID profiling[68]. See Supplementary Table 5, Tab 1-2 for details. **d,** Increased RNA binding by VIM at OMM upon PUR treatment. The relative APEX-PS-OMM enrichment (fold change of biotinylation) of +PUR vs –PUR samples was determined by comparing 129C&130N against 127C&128N. The fold change for each ribosome-independent OMM RBPs is shown and values represent means ± SD.

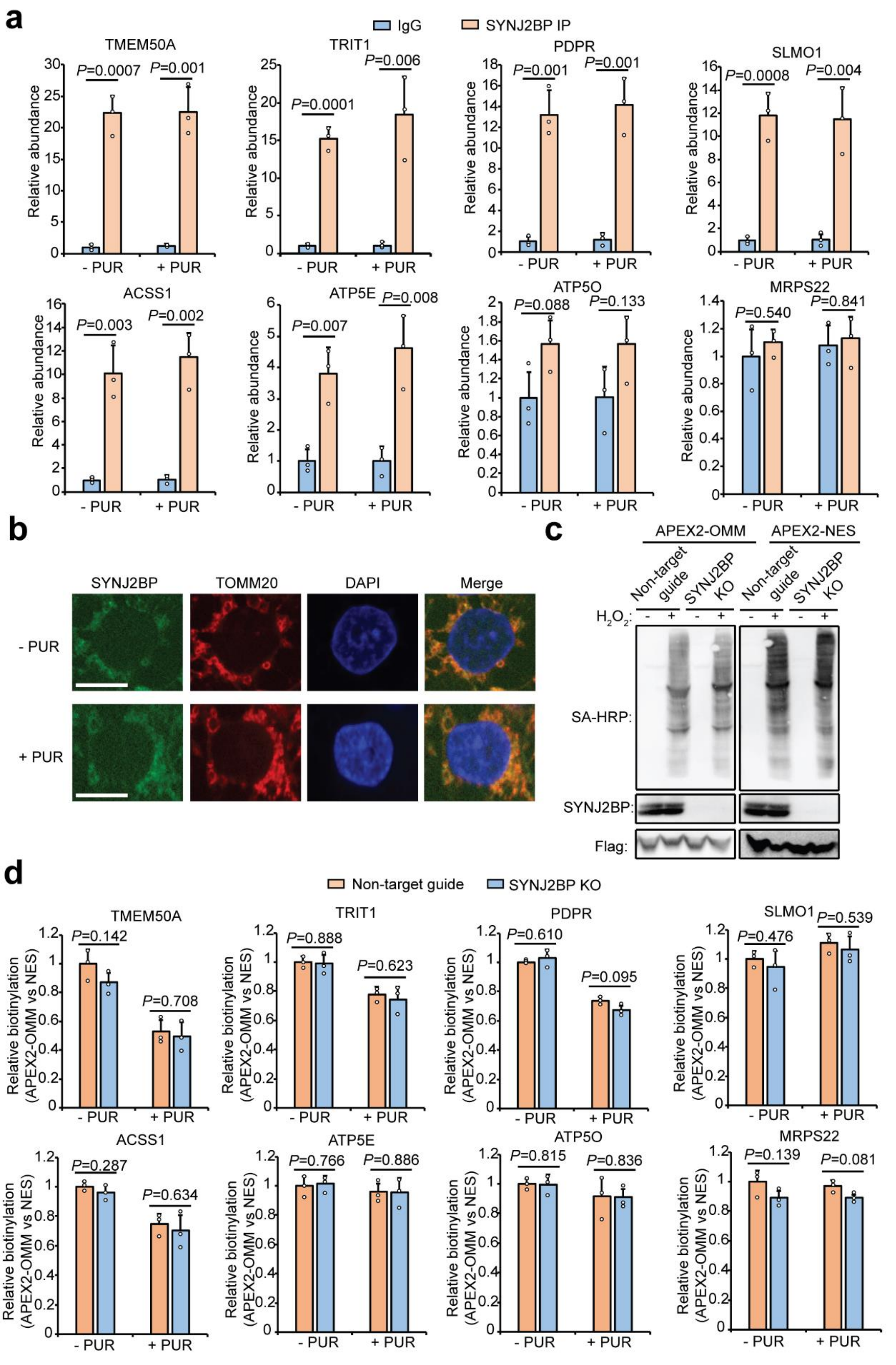

Supplementary Figure 10. Validation of SYNJ2BP's mRNA clients. a, Validation of RIP-seq identified SYBNJ2P mRNA clients. From the RIP-seq data shown in Supplementary Table 6, we selected 11 highly-enriched mitochondrial mRNAs (including 5 mRNAs shown in Fig. 6e) for validation by CLIP and qRT-PCR. CLIP

was performed both with and without PUR treatment. Negative controls are IARS2 (shown in Fig. 6e), ATP5O, MRPS22, OMM-localized mRNAs[11] that were not enriched by SYNJ2BP RIP-seq. **b,** Confocal imaging of endogenous SYNJ2BP with and without PUR treatment. Anti-TOM20 stains mitochondria and DAPI stains nuclei. Scale bars, 10 µm. **c,** Proximity labeling with biotin-phenol in wild-type and SYNJ2BP knockout HEK cells. **d,** Enrichment of specific mRNAs at the OMM by APEX RNA labeling, in wild-type HEK and SYNJ2BP KO cells, with and without PUR treatment. Negative controls are IARS2, ATP5O and MRPS22, OMM-localized mRNAs[11] that were not enriched by SYNJ2BP RIP-seq. Data for other five mRNA clients and IARS2 are shown in Fig. 6f. Two-sided Student's *t* test was performed and values represent means ± SD from three biological replicates.

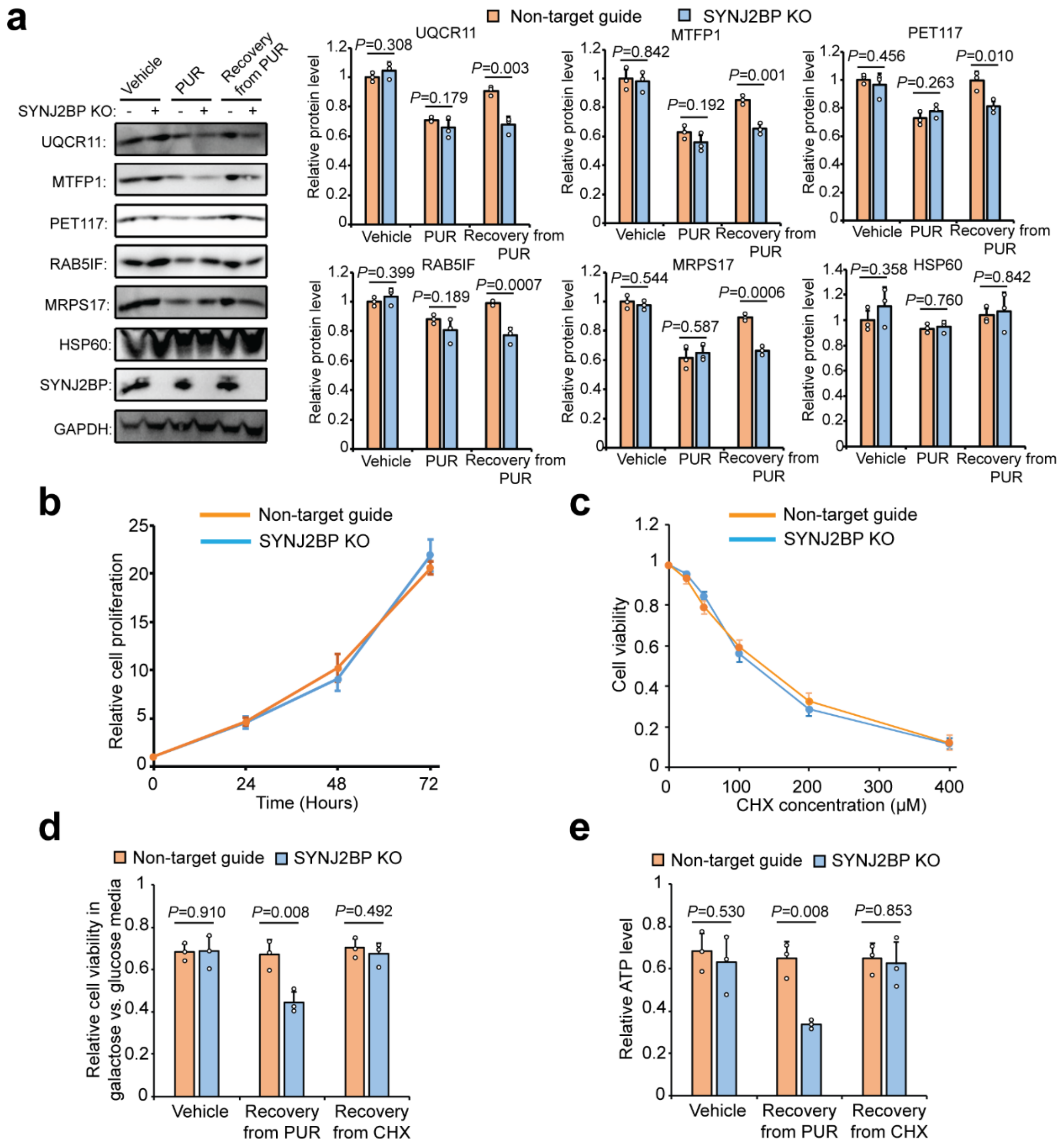

Supplementary Figure 11. SYNJ2BP promotes cellular recovery from PUR stress. a, Western blot analysis of protein levels before, after, and 12 hours after PUR treatment, in wild-type and SYNJ2BP KO HEK cells. UQCR11, MTFP1, PET117, RAB5IF and MRPS17 are validated mRNA clients of SYNJ2BP. HSP60 is a negative control - a mitochondrial protein whose mRNA is not a SYNJ2BP client. Right: quantification of western blot data from three biological replicates. **b,** Proliferation of wild-type versus SYNJ2BP KO HEK cells, using MTS assay. **c,** Same as (b) but following treatment of cells with cycloheximide (CHX). **d**, Preferential utilization of galactose versus glucose as a carbon source in SYNJ2BP knock-out cells. **e,** Measurement of ATP levels in SYNJ2BP KO cells pre-treated with CHX or PUR. Two-sided Student's *t* test was performed and values represent means ± SD from three biological replicates.

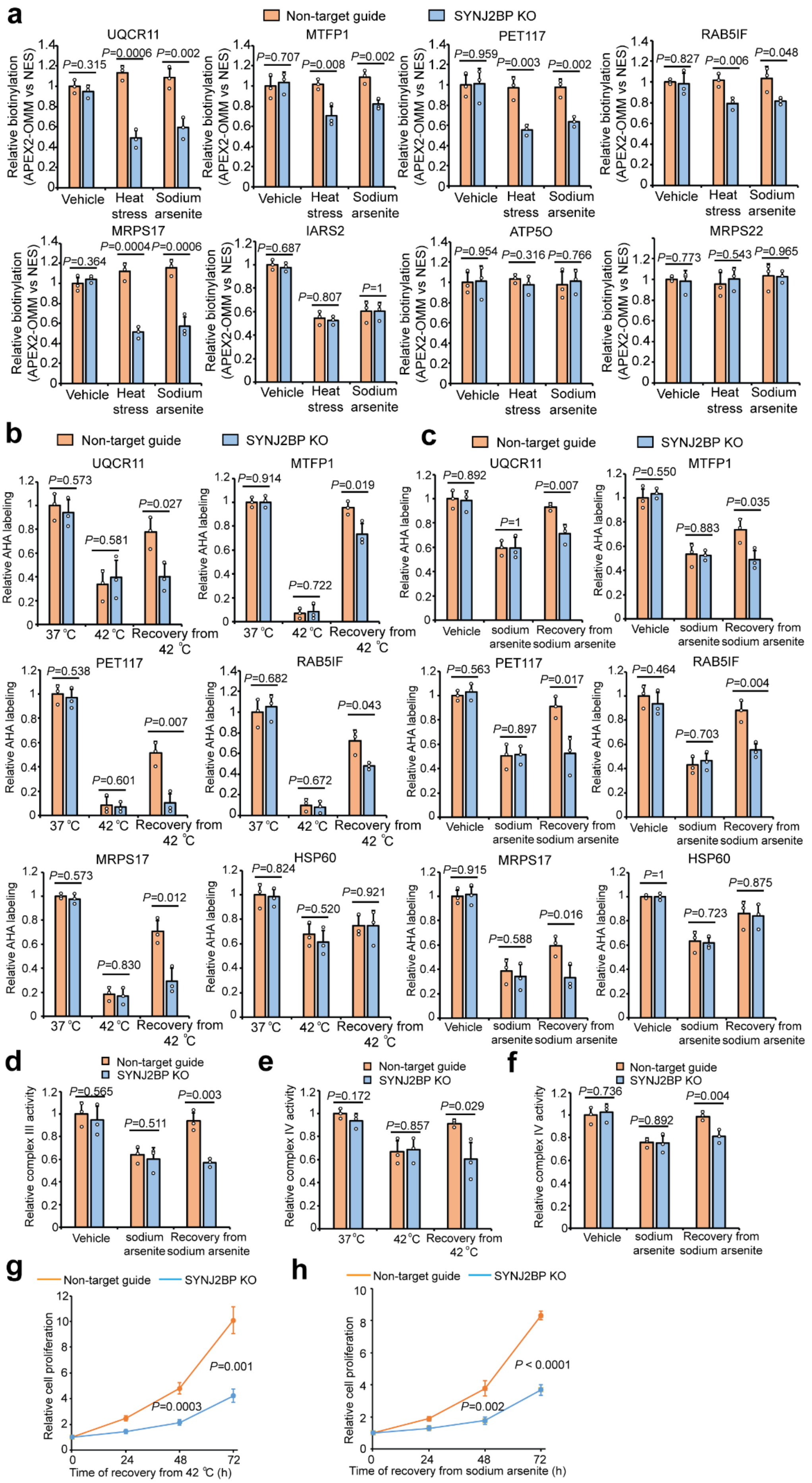
a
Non-target guide
SYNJ2BP KO
UQCR11
MTFP1
PET117
RAB5IF
MRPS17
IARS2
ATP5O
MRPS22
Relative biotinylation (APEX2-OMM vs NES)
Vehicle
Heat stress
Sodium arsenite
P=0.315
P=0.0006
P=0.002
P=0.707
P=0.008
P=0.002
P=0.959
P=0.003
P=0.002
P=0.827
P=0.006
P=0.048
P=0.364
P=0.0004
P=0.0006
P=0.687
P=0.807
P=1
P=0.954
P=0.316
P=0.766
P=0.773
P=0.543
P=0.965
b
Non-target guide
SYNJ2BP KO
UQCR11
MTFP1
PET117
RAB5IF
MRPS17
HSP60
Relative AHA labeling
37 °C
42 °C
Recovery from 42 °C
P=0.573
P=0.581
P=0.027
P=0.914
P=0.722
P=0.019
P=0.538
P=0.601
P=0.007
P=0.682
P=0.672
P=0.043
P=0.573
P=0.830
P=0.012
P=0.824
P=0.520
P=0.921
c
Non-target guide
SYNJ2BP KO
UQCR11
MTFP1
PET117
RAB5IF
MRPS17
HSP60
Relative AHA labeling
Vehicle
sodium arsenite
Recovery from sodium arsenite
P=0.892
P=1
P=0.007
P=0.550
P=0.883
P=0.035
P=0.563
P=0.897
P=0.017
P=0.464
P=0.703
P=0.004
P=0.915
P=0.588
P=0.016
P=1
P=0.723
P=0.875
d
Non-target guide
SYNJ2BP KO
Relative complex III activity
Vehicle
sodium arsenite
Recovery from sodium arsenite
P=0.565
P=0.511
P=0.003
e
Non-target guide
SYNJ2BP KO
Relative complex IV activity
37°C
42°C
Recovery from 42°C
P=0.172
P=0.857
P=0.029
f
Non-target guide
SYNJ2BP KO
Relative complex IV activity
Vehicle
sodium arsenite
Recovery from sodium arsenite
P=0.736
P=0.892
P=0.004
g
Non-target guide
SYNJ2BP KO
Relative cell proliferation
Time of recovery from 42 °C (h)
P=0.0003
P=0.001
h
Non-target guide
SYNJ2BP KO
Relative cell proliferation
Time of recovery from sodium arsenite (h)
P=0.002
P < 0.0001

Supplementary Figure 12. SYNJ2BP promotes cellular recovery from heat and sodium arsenite stresses. a, Enrichment of five SYNJ2BP-dependent mRNAs at the OMM by APEX RNA labeling, in wild-type HEK and SYNJ2BP KO cells, with and without heat or sodium arsenite treatment. Negative controls are IARS2, ATP5O and MRPS22, OMM-localized mRNAs[11] that were not enriched by SYNJ2BP RIP-seq. **b-c,** Quantification of protein synthesis for SYNJ2BP clients in SYNJ2BP knock-out cells under heat (**b**) and sodium arsenite (**c**) stresses. Typical Western blot results are shown in **Fig. 7e** and **Fig. 7f**. **d,** Evaluation of complex III activity in SYNJ2BP knock-out cells following sodium arsenite stress. **e-f,** Evaluation of complex IV activity in SYNJ2BP knock-out cells following heat (**e**) or sodium arsenite (**f**) stress. **g-h,** Evaluation of cell proliferation in SYNJ2BP knock-out cells during the recovery from heat (**g**) and sodium arsenite stress (**h**). Two-sided Student's *t* test was performed and values represent means ± SD from three biological replicates.

RT-qPCR primers	
UQCR11 forward	ctgggtcccgacggcctaca
UQCR11 reverse	ttatccttcttaaacttgcc
MTFP1 forward	agcgcgatctctaccgggac
MTFP1 reverse	tggccacgccatagctcagc
PET117 forward	ggagctcgaaggtggtgctg
PET117 reverse	caatgtctctgataactccg
RAB5IF forward	gattctgcctgatcaatgca
RAB5IF reverse	cataaacccttccttcgtga
MRPS17 forward	agactgctaaagtgagagtg
MRPS17 reverse	caatatccccaactgtgcac
TRIT1 forward	tgcacgagcagttcccgtgg
TRIT1 reverse	cgctggcctagctgcaacgc
TMEM50A forward	agagggcttgagatgctcag
TMEM50A reverse	catctatgataatccaccag
PDPR forward	tggaagacaaagagccagcc
PDPR reverse	cctccacagatgaccacctg
SLMO1 forward	gctcggagcacgtgtttggc
SLMO1 reverse	cgtccacgcggcgctgtagc
ACSS1 forward	gcagcacagccaggctcgta
ACSS1 reverse	cagtcccagacggtgtggta
ATP5E forward	gactcagctacatccgatac
ATP5E reverse	cgttgctgccagaagtcttc
IARS2 forward	actagtggaacaacacggca
IARS2 reverse	ggccaccaacctcagataaga
GAPDH forward	ttcgacagtcagccgcatcttctt
GAPDH reverse	gcccaatacgaccaaatccgttga

Supplementary Table 1. RT-qPCR primers used in this study.
