# Supplementary material for: Spatiotemporally-resolved mapping of RNA binding proteins via functional proximity labeling reveals a mitochondrial mRNA anchor promoting stress recovery
Source: Nat Commun. 2021 Aug 17;12:4980. doi: 10.1038/s41467-021-25259-2 (PMC8370977; doi:10.1038/s41467-021-25259-2)
Supplement: Supplementary file 9 — Description of additional supplementary files [file 41467_2021_25259_MOESM9_ESM.docx]

Description of additional supplementary items

Title: Supplementary Data 1.

Description: The 11-plex TMT proteomics results for assigning nuclear and nucleolar RBPs. HEK cells stably expressing nuclear APEX2-NLS or nucleolar APEX2-NIK3x were subjected to proximity biotinylation and FA crosslinking, as shown in Fig. 3a. The TMT ratios for all unfiltered proteins are shown in Tab1. For assigning nuclear RBPs by the pairwise ROC strategy, the true-positives (TPs) are literature-validated nuclear RBPs and the false-positives (FPs) are mitochondrial matrix proteins, which are listed in Tab 2. For each replicate, the proteins were first ranked in a descending order by TMT ratio (128N/126C, 128C/127N, 129N/127N – shaded grey in Tab 1). Through ROC analysis, the cutoff was set at the rank position where TP rate - FP rate (TPR-FPR) was maximal. The three independently-filtered datasets were intersected. For determining the nucleolar RBPome by the pairwise ROC approach, the TPs listed in Tab3 are the known RBPs from OOPS datasets with nucleolar annotation according to GOCC. The FPs were non-nuclear proteins without OOPS RBP annotation, also listed in Tab 3. The proteins from Tab 1 were first ranked in descending order by TMT ratio. For each replicate, we analyzed two ratios: 130C/126C and 130C/128N for replicate 1; 131N/129C and 131N/128C for replicate 2; and 131C/129C and 131C/129N for replicate 3 (shaded light blue in Tab 1). We then performed ROC-based filtering as described above. Potential glycosylated proteins were removed from the dataset manually, according to their annotation as glycoproteins or exclusively secretory pathwaylocalized (e.g. ER/Golgi lumen, plasma membrane, extracellular regions). Column definitions in Tab 4.

Title: Supplementary Data 2.

Description: The list of nuclear RBPs. Nuclear RBPome1 determined by the pairwise ROC approach is listed in Tab 1. In Tab 1, our nuclear RBPome is also compared to 11 previous RBP profiling datasets (references shown in Tab 5). RBPs identified by at least one dataset were assigned as “known RBPs” and the remaining ones were assigned as RBP “orphans”. The RBPs identified by oligodT pulldown-based methods are assigned as poly (A) RBPs and the remaining ones are assigned as non-poly (A) RBPs. The types of RNA binding with non-poly (A) RBPs are manually annotated based on literature. The domains of RBPs were obtained from Pfam and the RBPs containing at least one classic RBD were assigned as “RBPs with classic RBDs”. The RBPs containing only non-classical RBDs were assigned as “RBPs with non-classic RBDs”. Nuclear RBPome2 determined by statistical analysis is listed in Tab 2 with fold-change and adjusted p-values. Tab 3 shows the previously annotated nuclear proteins and RBPs used for nuclear RBPome specificity analysis in Fig. 3f, 3g, 4f, 4g and Supplementary Fig.5e-f. Tab 4 shows the gold standard nuclear RBPs used for sensitivity analysis in Fig. 3h and Supplementary Fig.5g. Tab 5 shows the manually removed glycoproteins. Column definitions in Tab 6.

Title: Supplementary Data 3.

Description: The list of nucleolar RBPs. Nucleolar RBPs determined by the pairwise ROC approach are listed in Tab 1. In Tab 1, our nucleolar RBPs were compared to 11 previous RBP profiling datasets (references shown in Tab 4). RBPs identified by at least one dataset were assigned as “known RBPs” and the remaining ones were assigned as RBP “orphans”. The RBPs identified by oligodT pulldown-based methods are assigned as poly (A) RBPs and the remaining ones are assigned as non-poly (A) RBPs. The types of RNA binding with non-poly (A) RBPs are manually annotated based on literature. The domains of RBPs were obtained from Pfam. Nucleolar RBPs generated from statistical analysis are listed in Tab 2 with fold change and adjusted p values. Tab 3 shows the gold standard list of well-established nucleolar RBPs used for sensitivity analysis in Supplementary Fig. 8g. Tab 4 shows the manually removed glycoproteins. Column definition is shown in Tab 5.

Title: Supplementary Data 4.

Description: The 11-plex TMT proteomics results for assigning OMM-localized RBPs. HEK cells stably expressing APEX2-OMM were treated with puromycin or left untreated, then subjected to proximity biotinylation and FA crosslinking as shown in Fig. 5a. The TMT ratios for all unfiltered proteins are shown in Tab 1. For each replicate, the APEX-PSOMM sample was compared to both a negative control omitting H2O2 and an APEX-PS-NES sample. To compare APEX-PS-OMM samples to background controls, a curated list of known OMM proteins62 was used as TPs (TP1, tab 2) and mitochondrial matrix proteins annotated by GOCC were assigned as FPs (FP1, tab 2). To compare APEX-PS-OMM samples to APEX-PS-NES reference controls, the same set of known OMM proteins was used as TPs (TP2, tab 2) and cytosolic proteins without mitochondrial annotation according to GOCC were assigned as FPs (FP2, tab 2). The proteins were first ranked in descending order by TMT ratio, and cutoff was determined by pairwise ROC analysis as described above. For assignment of OMM RBPs under basal conditions, proteins above the 127C/126C and 127C/131N cutoffs (replicate 1) were intersected with proteins above the 128N/126C and 128N/131N cutoffs (replicate 2) (shaded grey in Tab 1). For assignment of OMM RBPs following PUR treatment, proteins above the 129C/128C and 129C/131C cutoffs (replicate 1) were intersected with proteins above 130N/128C and 130N/131C cutoffs (replicate 2) (shaded light blue in Tab 1). Glycosylated proteins were manually removed from the final lists. Column definitions in Tab 3.

Title: Supplementary Data 5.

Description: The lists of OMM-localized RBPs under basal and puromycin (PUR)-treated conditions. The final OMM-localized RBPs under basal and PUR conditions are listed in Tab 1 and Tab 2, respectively. In Tabs 1 and 2, our OMM RBPs were compared to 11 previous RBP profiling datasets (references shown in Tab 3). RBPs identified by at least one dataset were assigned as “known RBPs” and the remaining ones were assigned as RBP “orphans”. The RBPs identified by oligodT pulldown-based methods are assigned as poly (A) RBPs and the remaining ones are assigned as non-poly (A) RBPs. The types of RNA binding with non-poly (A) RBPs are manually annotated based on literature. The domains of RBPs were obtained from Pfam. The OMM RBPs are compared with OMM proteins annotated by APEX2-OMM profiling62. The sub-mitochondrial annotation was obtained from GOCC, mitochondrial annotation was obtained from GOCC, mitoCarta and literature. Proteins involved in mitochondrial-ER contacts were annotated by split-TurboID profiling68. Tab 3 shows the manually removed glycoproteins. Column definitions in Tab 4.

Title: Supplementary Data 6.

Description: List of SYNJ2BP mRNA clients. The mRNA clients of SYNJ2BP as detected by RIP-seq are listed in Tab 1. The SYNJ2BP RIP was performed with a SYNJ2BP antibody along with an IgG negative control. The relative enrichment of each mRNA was obtained from the fold change of gene-level FPKM (fragments per kilobase of transcript per million mapped reads) values between SYNJ2BP IP and IgG samples. The mRNA clients validated by CLIP-qPCR are shown. Column definitions in Tab 2.
